# STARZ Neonatal AKI Risk Stratification Cut-off Scores for Severe AKI and Need for Dialysis in Neonates

**DOI:** 10.1016/j.ekir.2022.06.020

**Published:** 2022-07-14

**Authors:** Sidharth Kumar Sethi, Rupesh Raina, Sanjay Wazir, Gopal Agrawal, Ananya Vadhera, Nikhil Nair, Kritika Soni, Abhishek Tibrewal, TINKER Working Group, Naveen Bajaj, Naveen Parkash Gupta, Shishir Mirgunde, Jagdish Sahoo, Binesh Balachandran, Kamran Afzal, Anubha Shrivastava, Jyoti Bagla, Sushma Krishnegowda, Ananth Konapur

**Affiliations:** 1 Pediatric Nephrology, Kidney Institute, Medanta, The Medicity Hospital, Gurgaon, Haryana 122001, India; 2 Akron Children’s Hospital, Akron, Ohio, USA; 3 Department of Neonatology, Cloudnine Hospital, Gurgaon, Haryana 122001, India; 4 Maulana Azad Medical College, New Delhi, India; 5 Case Western Reserve University School of Medicine, Cleveland, Ohio, USA; 6 Akron Nephrology, AGMC Cleveland Clinic, Cleveland, Ohio, USA

**Keywords:** acute kidney injury, neonates, neonatal acute kidney injury, pediatrics, peritoneal dialysis, STARZ neonatal score

## Introduction

Neonatal acute kidney injury (AKI) is a significant pathology associated with higher mortality rates, longer neonatal intensive care stay, and worse clinical outcomes.^1^^,^^2^ In order to mitigate the avoidable outcomes, it is important to identify AKI early and start early therapeutic measures.^2^^,^^3^ There have been previous attempts to derive illness severity scores, such as the Clinical Risk Index for Babies, the Simplified age-weight-sex score, Pediatric Risk of Mortality and Pediatric Index of Mortality among neonates and children.^4^ These scores assess the illness severity, and cannot be used for the risk stratification for AKI or mortality. Risk of mortality in low birth weight neonates has been predicted by the NMR-2000 score, which was validated for use in low to middle income countries.^5^ A specific score for AKI risk stratification in older children, Renal Angina Index, which uses the reduction in estimated creatinine clearance, fluid balance and high-risk disease states, has been shown to predict AKI accurately among various high-risk disease states.^6^ Neonatal AKI risk scores are imperative to help predict which neonates are at a high-risk and should have early directed interventions. The STARZ score predicts the risk of AKI in neonates with high sensitivity (92.8%), specificity (87.4%), positive predictive value (80.5%), negative predictive value (95.6 %,) and accuracy (89.4%), which allows for its validation for use in low to middle income countries to facilitate the rapid identification of at-risk neonates.^7^^,^^8^ The variables of the STARZ score are shown in Table 1. This research letter reports cut-off scores required for identifying risk of severe AKI and dialysis need in neonates. The methodology and statistical analysis of the study is provided in the Supplementary Material.

**Table 1:** STARZ scoring model

Variables		Assigned score	
Age at entry in NICU (hs)	<25.5	6	
	≥25.5	0	
PPV in the delivery room	Yes	7	
	No	0	
Gestational age (wks)	<28	7	
	≥28	0	
Sepsis (during the NICU stay)	Yes	6	
	No	0	
Significant cardiac disease	Yes	10	
	No	0	
Urine output^a^ (ml/kg/h)	<1.32	7	
	≥1.32	0	
Serum creatinine^a^ (mg/dl)	≥0.98	20	
	<0.98	0	
Use of nephrotoxic drugs	Yes	11	
	No	0	
Use of furosemide	Yes	9	
	No	0	
Use of inotropes	Yes	17	
	No	0	

AKI, acute kidney disease; NICU, neonatal intensive care unit; PPV, positive pressure ventilation.

Nephrotoxic drugs included Vancomycin or Colistin or Amphotericin B

Significant cardiac disease included hemodynamically significant patent ductus arteriosus, persistent pulmonary hypertension of the newborn, cardiogenic shock and other congenital heart disease

Inotropes included Dopamine or Dobutamine or Epinephrine or Norepinephrine

a First 12 hours post admission in NICU.

## Results

The current study included 1005 neonates (646 without AKI and 359 with AKI) that met the inclusion criteria. The flow of the study is shown in Supplementary Figure S1. Of the 359 neonates with AKI, 58 (16.2%) had stage 1 AKI, 77 (21.4%) had stage 2 AKI, and 224 (62.4%) had stage 3 AKI. The neonates with gestational age at birth less than 28 weeks were 34 (3.4%), those who required positive pressure ventilation in delivery room were 189 (18.8%), those who were aged less than 25.5 hours at entry in neonatal intensive care unit (NICU) were 580 (57.7%), those who had sepsis during NICU stay were 684 (68.1%), and those with significant cardiac disease were 288 (28.7%). Neonates whose serum creatinine was greater than or equal to 0.98 mg/dl were 314 (31.2%), those with urine output less than 1.32 ml/kg/h were 512 (50.9%), those with nephrotoxic drug use were 920 (91.5%), those with furosemide use 44 (4.4%), and those with inotrope use were 388 (38.6%). A total of 52 (5.2%) neonates died during NICU stay. The median (interquartile range [IQR]) STARZ score was 34 (23–57) and length of NICU stay was 10 (5–18) days (Supplementary Table S1).

**Figure 1:**
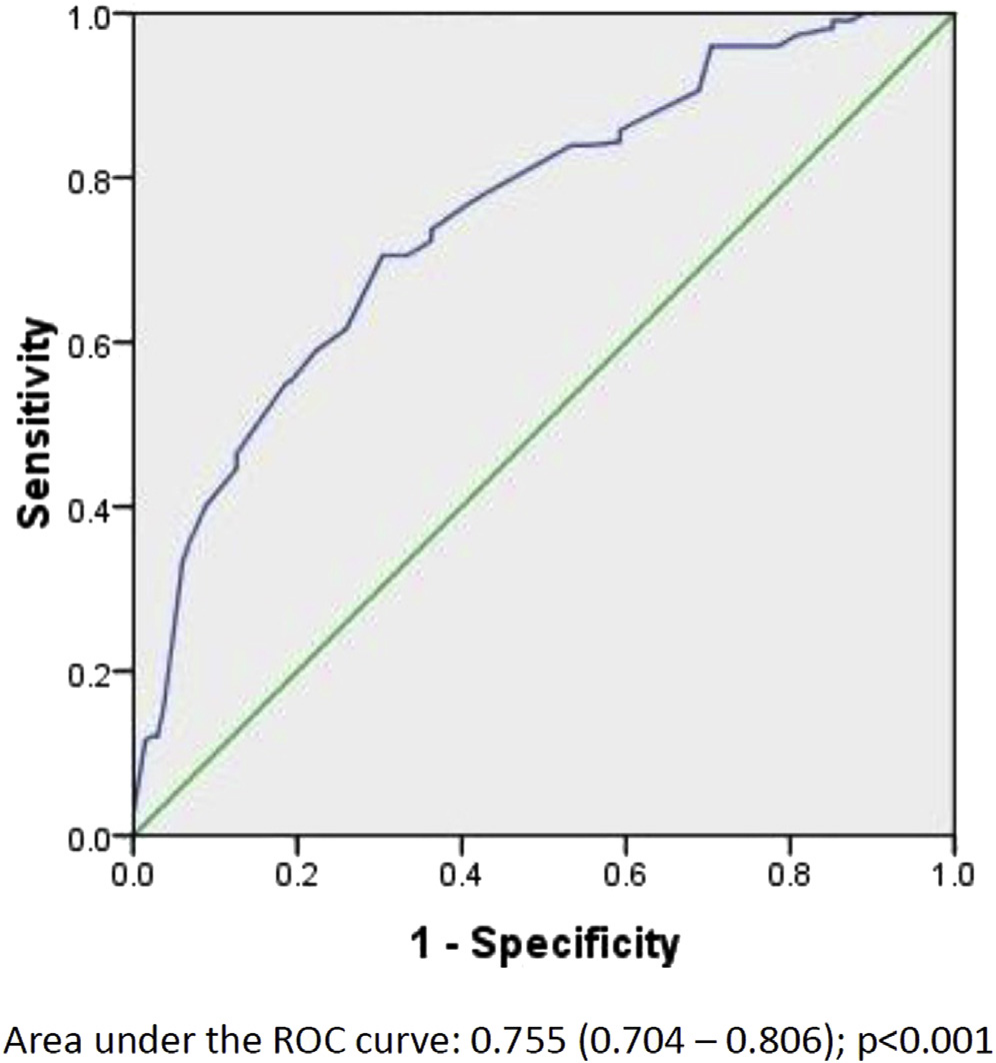
Area under the receiver operating characteristic for severe neonatal acute kidney injury

The comparison of different variables among neonates with severe AKI (AKI stage 3) versus mild to moderate AKI (AKI stages 1–2) is presented in Table 2. Comparing these 2 groups of neonates, respectively, the proportion with significant cardiac disease (107 [47.8%] vs. 39 [28.9%], *P* < 0.001), inotropes usage (162 [72.3%] vs. 82 [60.7%], *P* = 0.027), and those with serum creatinine greater than or equal to 0.98 mg/dl (223 [99.6%] vs. 71 [52.6%], *P* < 0.001) were significantly higher among those with severe AKI than among those mild to moderate AKI. The median (IQR) STARZ score was found to be significantly higher (67 [54–77] vs. 50 [40–61]. *P* < 0.001) among neonates with severe AKI than among those with mild to moderate AKI. The best cut-off value STARZ score for severe AKI was found to be 59 with a sensitivity of 71% and specificity of 70% and with an area under the receiver operating characteristic curve of 0.755 (95% CI: 0.704 – 0.806, *P* < 0.001) (Figure 1). The median (IQR) time to AKI was observed to be significantly lower (1 [1–3] vs. 3 [1–3] days, *P* < 0.001) among neonates with severe AKI than among those with mild to moderate AKI.

**Table 2:** Comparison of different variables among neonates without AKI versus stage 1 AKI versus stage 2 AKI versus stage 3 AKI

Variables	No AKI (*n* = 646)	Stage 1 (*n* = 58)	Stage 2 (*n* = 77)	Stage 3 (*n* = 224)	*P* value
Maternal antenatal characteristics [Y]	184 (28.5%)	22 (37.9%)	36 (46.8%)	100 (44.6%)	<0.001
Severe peripartum event [Y]	7 (1.1%)	1 (1.7%)	2 (2.6%)	4 (1.8%)	0.386
Site of delivery (Outborn)	254 (39.3%)	24 (41.4%)	32 (41.6%)	118 (52.7%)	0.007
Mode of delivery (Cesarean)	377 (58.4%)	39 (67.2%)	52 (67.5%)	120 (53.6%)	0.084
Gender (Male)	446 (69%)	42 (72.4%)	54 (70.1%)	155 (69.2%)	0.967
Gestational age birth (<28 wks)	15 (2.3%)	0 (0%)	6 (7.8%)	13 (5.8%)	0.005
Birth weight (<1000 gm)	17 (2.6%)	0 (0%)	5 (6.5%)	21 (9.4%)	<0.001
PPV in delivery room [Y]	91 (14.1%)	11 (19%)	21 (27.3%)	66 (29.5%)	<0.001
Age at NICU entry (<25.5 hs)	328 (50.8%)	42 (72.4%)	51 (66.2%)	159 (71%)	<0.001
APGAR score at 5 mins^a^	8 (7–8)	8 (7–8)	7 (6–8)	7 (6–8)	<0.001
Respiratory support in NICU	414 (64.1%)	50 (86.2%)	69 (89.6%)	195 (87.1%)	<0.001
Sepsis during the NICU stay [Y]	379 (58.7%)	40 (69%)	71 (92.2%)	194 (86.6%)	<0.001
Significant cardiac disease	142 (22%)	15 (25.9%)	24 (31.2%)	107 (47.8%)	<0.001
Necrotizing enterocolitis [Y]	16 (2.5%)	2 (3.4%)	5 (6.5%)	16 (7.1%)	0.008
Intraventricular hemorrhage [Y]	21 (3.3%)	3 (5.2%)	8 (10.4%)	17 (7.6%)	0.005
Any surgical intervention [Y]	32 (5%)	3 (5.2%)	4 (5.2%)	16 (7.1%)	0.65
Evidence of fluid overload^b^ [Y]	7 (1.1%)	0 (0%)	1 (1.3%)	6 (2.7%)	0.27
Multiple seizure^b^ [Y]	55 (8.5%)	7 (12.1%)	6 (7.8%)	46 (20.5%)	<0.001
Nephrotoxic drug [Y]	564 (87.3%)	57 (98.3%)	77 (100%)	222 (99.1%)	<0.001
Furosemide [Y]	23 (3.6%)	1 (1.7%)	4 (5.2%)	16 (7.1%)	0.111
Caffeine [Y]	111 (17.2%)	6 (10.3%)	23 (29.9%)	62 (27.7%)	<0.001
Inotropic drugs [Y]	144 (22.3%)	29 (50%)	53 (68.8%)	162 (72.3%)	<0.001
Mean arterial pressure^a^^,^^b^	51 (44–67)	49 (43–56)	46 (42–57)	46 (41–54)	<0.001
IV fluid intake (ml/kg/d)^a^^,^^b^	60 (60–68)	60 (60–69)	60 (60–65)	60 (60–70)	0.184
Creatinine ≥0.98 mg/dl [Y]	20 (3.1%)	33 (56.9%)	38 (49.4%)	223 (99.6%)	<0.001
Urine output <1.32 ml/kg/hr [Y]	292 (45.2%)	32 (55.2%)	45 (58.4%)	143 (63.8%)	<0.001
Serum urea (mg/dl)^a^^,^^b^	20 (17–24)	38 (32–42)	28 (23–38)	42 (35–53)	<0.001
Serum sodium (meq/l)^a^^,^^b^	133 (132–138)	138 (134–142)	135 (130–139)	135 (132–139)	<0.001
Serum potassium(meq/l)^a^^,^^b^	4.5 (4.2–4.7)	5.2 (5–5.4)	4.8 (4.7–5.2)	5.3 (5–5.5)	<0.001
Hb (g/l)^a^^,^^b^	16.5 (15.4–18.4)	17 (15.8–19)	17.2 (16–18.2)	16.2 (14.7–17.5)	0.016
Serum pH^a^^,^^b^	7.32 (7.28–7.32)	7.32 (7.25–7.41)	7.29 (7.28–7.32)	7.26 (7.2–7.32)	<0.001
STARZ score^a^	24 (17–34)	47 (37–60)	50 (43–65)	67 (54–77)	<0.001
No. of days to AKI^a^	-	3 (2–7)	3 (1–3)	1 (1–3)	<0.001
NICU stay (ds)^a^	8 (4.75–15.25)	10 (6–17.25)	18 (9–30.75)	11 (5–22)	<0.001
Death	10 (1.5%)	1 (1.7%)	2 (2.6%)	39 (17.4%)	<0.001

AKI, acute kidney injury; Hb, hemoglobin; IV, Intravenous; IQR, interquartile range; NICU, neonatal intensive care unit; PPV, positive pressure ventilation; Y, yes.

Nephrotoxic drugs included Vancomycin or Colistin or Amphotericin B

Inotropes included Dopamine or Dobutamine or Epinephrine or Norepinephrine

Significant cardiac disease included patent ductus arteriosus, pulmonary hypertension of the newborn, ventricular septal defect; shock

Severe peripartum event included cord prolapsed, precipitate labor, abruption

Multiple seizures were defined as >1 seizure episode in the first 12 h

Fluid overload defined as >10% during the first 12 h post admission

Even a single exposure of the drug has been considered as usage of drug

Maternal characteristics recorded were- maternal diabetes, maternal pregnancy induced hypertension, maternal bacterial/ viral infections/ IUGR/ oligohydramnios/ polyhydramnios/ use of drugs during pregnancy (ACE-inhibitors, NSAIDs, tobacco, alcohol, antidepressants, steroids)

a Reported as median (IQR); for others as proportion

b First 12 hours post admission in NICU

The comparison of different variables among stage 3 AKI neonates treated with peritoneal dialysis (PD) versus those treated without PD is presented in Supplementary Table S2. The proportion of neonates with gestational age at birth less than 28 weeks (5 [14.7%] vs. 8 [4.2%], *P* = 0.031], with significant cardiac disease (28 [82.4%] vs. 79 [41.6%], *P* < 0.001), with furosemide usage (6 [17.6%] vs. 10 [5.3%], *P* = 0.02), with inotropes usage (33 [97.1%] vs. 129 [67.9%], *P* < 0.001], and with urine output less than 1.32 ml/kg/h (30 [88.2%] vs. 113 [59.5%], *P* = 0.001) were significantly higher among those with severe AKI than among those with mild to moderate AKI. As expected, the median (IQR) STARZ score was observed to be significantly higher (77 [71–84] vs. 64 [50 – 77], *P* < 0.001) among stage 3 AKI neonates treated with PD than among those treated without PD. The best cut-off value STARZ score was found to be 66 with a sensitivity of 97% and specificity of 52% for PD use with an area under the receiver operating characteristic curve of 0.804 (95% CI: 0.738 –0.870), *P* < 0.001) (Supplementary Figure S2). The median (IQR) time to AKI was observed to be significantly lower (1 [1–2] vs. 1 [1–3] days, *P* = 0.017) among stage 3 AKI neonates treated with PD than among those treated without PD.

Mortality was observed to be significantly higher among those with severe AKI than among those with mild to moderate AKI (39 [17.4%] vs. 3 [2.2%], odds ratio [95% CI]: 9.28 [2.81–30.65]), and significantly higher among stage 3 AKI neonates treated with PD than among those treated without PD (30 [88.2%] vs. 9 [4.7%], 150.83 [43.67–521.0). Nevertheless, the median (IQR) duration of stay in the NICU was significantly lower among those with severe AKI than among those with mild to moderate AKI (11 [5–22] vs. 14 [7–25] days. *P* = 0.033); and significantly lower among stage 3 AKI neonates treated with PD than among those treated without PD (7 [3–11] vs. 12 [6–23] days. *P* = 0.001) (Table 2 and Supplementary Table S2).

To summarize, we found the following cut-offs for neonatal AKI prediction: STARZ score less than 31.5 predicts low probability of AKI; STARZ score less than 59 predicts low probability of severe AKI, and STARZ score less than 66 predicts low probability of severe AKI with the need for PD. The cut-off scores were found to increase with increased AKI severity. Similar studies to derive cut-offs to predict severe AKI and need for dialysis have been done with urine neutrophil gelatinase-associated lipocalin at admission in adults.^9^ To our knowledge, this is the first of its kind study to use a scoring system that can easily be replicated in NICU. Nevertheless, further studies are needed to validate the cut-off scores. These cut-offs can help a clinician to determine the need for dialysis requirement, anticipate severe neonatal AKI and acts as a beneficial and easy clinical adjunct to neonatal intensive care units of all types.

## Disclosure

All the authors declared no competing interests.

## Author Contributions

All authors made substantial contributions to conception and design, acquisition of data, analysis and interpretation of data; drafting the article or revising it critically for important intellectual content. All authors gave final approval of the version to be published.

## Appendix

### List of the TINKER Working Group

Naveen Bajaj, Neonatology, Deep Hospital, Ludhiana, Punjab, India

Naveen Parkash Gupta, Neonatology, Madhukar Rainbow Children’s Hospital, New Delhi, India

Shishir Mirgunde, Government Medical College, Miraj, Maharashtra, India

Jagdish Sahoo, Department of Neonatology, IMS & SUM Hospital, Bhubaneswar, India

Binesh Balachandran, Aster Mims hospital, Kottakkal, Kerala, India

Kamran Afzal, Department of Pediatrics, Jawaharlal Nehru Medical College, Aligarh, Aligarh Muslim University, Uttar Pradesh, India

Anubha Shrivastava, MLM Medical College, Prayagraj, Uttar Pradesh, India

Jyoti Bagla, ESI Post Graduate Institute of Medical Science Research, Basaidarapur, New Delhi, India

Sushma Krishnegowda, JSS Hospital, JSS Academy of Higher education and Research, Mysuru, Karnataka, India

Ananth Konapur, KIMS Hospital, Kurnool, Andhra Pradesh, India

## Supplementary Material

Supplementary File (PDF)

**Figure S1.** Flow of the study

**Figure S2.** Area under the ROC curve for stage 3 AKI with peritoneal dialysis

**Table S1.** Demographic profile of the neonates included in the study

**Table S2.** Comparison of different variables among stage 3 AKI neonates with versus without peritoneal dialysis
