# Supplementary material for: STARZ Neonatal AKI Risk Stratification Cut-off Scores for Severe AKI and Need for Dialysis in Neonates
Source: Kidney Int Rep. 2022 Jul 14;7(9):2108–11. doi: 10.1016/j.ekir.2022.06.020 (PMC9459073; doi:10.1016/j.ekir.2022.06.020)
Supplement: Supplementary File (PDF) [file mmc1.pdf]

Methodology

Study Design: The TINKER study is a multicentre, national, prospective database enrolling neonates from 11 centres across India. The original STARZ score was derived from the neonatal database on babies enrolled from August 2018-2019. In the present study, neonates from all the collaborative 11 centres across India from September 2019-August 2021 were enrolled.

Inclusion Criteria: Admitted neonates who need intravenous fluids for at least 48 hours

Exclusion Criteria:

- Mortality within 48 hours of admission
- Neonates not on any intravenous access
- Lethal anomaly including trisomy 13, 18 and anencephaly
- Neonates needed congenital cardiac surgery within first week of life, since these neonates are referred.

Data entry points

All the variables were collected daily during hospitalisation in the first week. After the first week, the initial value for each week was collected (for serum creatinine, all values were recorded regardless of the day of life/admission) till the end points were reached.

Data Collection

Demographic details (related to birth history and NICU admission), maternal demographic details, and detailed NICU admission data was collected in the online database. Detailed information regarding the AKI episodes and need for renal replacement therapy was also recorded. KDIGO criteria were used for AKI staging in neonates. Serum creatinine was recorded daily in AKI cases using the enzymatic method till the end points. The detailed methodology is provided in the TINKER database methodology paper and our previous STARZ derivation and validation publications.

End Points

Data was collected in all neonates until they were either discharged, transferred out of NICU, expired, or became 120 days of age.

Statistical analysis

The STARZ score delineates AKI risk in the first 7 days post admission and is calculated any time after 12 hours of admission in NICU. The score derived from our previous multicentre study included 10 variables with significant AKI association (7 categorical, 3 continuous). Each variable is assigned a score according to previously detailed methodology, with a score of 0 being assigned to reference group; the total score can range from 0 to100 and a cut off score ≥31.5 associated with high probability of AKI [Supplementary Table 1].

In this study, all the variables were tested for normality using Kolmogorov-Smirnov test. Categorical variables are summarized as frequencies and percentages, while continuous variables as medians and inter-quartile range (IQR; 25th to 75th percentiles). The following analyses were reported in this study: a) between neonates with stage 3 and stage 2 / 1 AKI; b) between stage 3 AKI neonates with and without peritoneal dialysis. The demographic, clinical variables and STARZ score in these groups were compared using the chi-square / Fischer exact test and the Wilcoxon's rank-sum / Kruskal Wallis test. The best cut-off value for the STARZ score was derived having maximum accuracy and minimal weighted error. The standard statistical measures [sensitivity, specificity, and area under the receiver operating characteristic (ROC) curve] were also reported for the STARZ score. A two-sided p value <0.05 has been considered to be statistically significant. Statistical software (SPSS version 20) was used for the statistical analyses.

Supplementary Table 1: Demographic profile of the neonates included in the study (n=1,005)

Variables	N (%) or Median (IQR)	
Maternal antenatal characteristics [Y]	342 (34%)	
Severe peri-partum event [Y]	14 (1.4%)	
Site of delivery [Outborn]	428 (42.6%)	
Mode of delivery [Caesarean]	588 (58.5%)	
Gender [Male]	697 (69.4%)	
Birth weight (<1,000 g)	43 (4.3%)	
Gestational age at birth (<28 weeks)	34 (3.4%)	
PPV in delivery room [Y]	189 (18.8%)	
Age at entry in NICU (hours)#	1005	19 (6 - 79)
Age at entry in NICU (<25.5 hours)	580 (57.7%)	
APGAR score at 5 minutes#	1005	8 (7 - 8)
Respiratory support in NICU [Y]	728 (72.4%)	
Sepsis during the NICU stay [Y]	684 (68.1%)	
Significant cardiac disease [Y]	288 (28.7%)	
Necrotizing Enterocolitis [Y]	39 (3.9%)	
Intraventricular hemorrhage [Y]	49 (4.9%)	
Any surgical intervention [Y]	55 (5.5%)	
Evidence of fluid overload* [Y]	14 (1.4%)	
Multiple Seizure* [Y]	114 (11.3%)	
Nephrotoxic drug [Y]	920 (91.5%)	
Furosemide [Y]	44 (4.4%)	
Caffeine [Y]	202 (20.1%)	
Inotropes [Y]	388 (38.6%)	
Mean arterial pressure*#	536	49 (43 - 63)
IV fluid intake (ml/kg/d)*#	977	60 (60 - 68)
Urine output <1.32 ml/kg/hr [Y]	512 (50.9%)	
Serum Urea (mg/dl)*#	645	23 (18 - 36)
Serum creatinine ≥0.98 mg/dl [Y]	314 (31.2%)	
Serum Sodium (meq/L)*#	749	134 (132 - 138)
Serum Potassium(meq/L)*#	750	4.6 (4.3 - 5.2)
Hb (g/L)*#	812	16.4 (15.4 - 18.2)
Serum pH*#	391	7.3 (7.26 - 7.32)
STARZ score#	1005	34 (23 - 57)
No. of days to AKI#	359	2 (1 - 3)
NICU stay (days)#	903	10 (5 - 18)
Death	52 (5.2%)	

#reported as median (IQR); for others as proportion

*First 12 hours post admission in NICU

IQR: Interquartile range; AKI: Acute Kidney Injury; NICU: Neonatal Intensive Care Unit; hr: hour; mg: milligram; dl: decilitre; cm: centimetre; ml: millilitre; L: Liter; Hb: haemoglobin; g: gram; IV: Intravenous; hr: hour; kg: kilogram; d: day; meq: milliequivalent; PPV: Positive pressure ventilation; Y: Yes

- Nephrotoxic drugs included Vancomycin or Colistin or Amphotericin B
- Inotropes included Dopamine or Dobutamine or Epinephrine or Norepinephrine
- Significant cardiac disease included PDA: Patent ductus arteriosus; PPHN: pulmonary hypertension of the newborn; VSD: Ventricular septal defect; shock
- Severe peripartum event included cord prolapsed, precipitate labour, abruption

Supplementary Table 2: Comparison of different variables among stage 3 AKI neonates with versus without peritoneal dialysis

Variables	Peritoneal dialysis		p value
	Yes (n=34)	No (n=190)	
Maternal antenatal characteristics [Y]	18 (52.9%)	82 (43.2%)	0.291
Severe peri-partum event [Y]	2 (5.9%)	2 (1.1%)	0.110
Site of delivery (Outborn)	15 (44.1%)	103 (54.2%)	0.278
Mode of delivery (Caesarean)	20 (58.8%)	100 (52.6%)	0.505
Gender (Male)	26 (76.5%)	129 (67.9%)	0.420
Birth weight (<1,000 gm)	7 (20.6%)	14 (7.4%)	0.024
Gestational age at birth (<28 weeks)$	5 (14.7%)	8 (4.2%)	0.031
PPV in delivery room [Y]$	14 (41.2%)	52 (27.4%)	0.104
Age at entry in NICU (<25.5 hours)$	27 (79.4%)	132 (69.5%)	0.240
APGAR score at 5 minutes#	7 (6 - 8)	7 (6 - 8)	0.08
Respiratory support in NICU	34 (100%)	161 (84.7%)	0.01
Sepsis during the NICU stay [Y]$	32 (94.1%)	162 (85.3%)	0.271
Significant cardiac disease [Y]$	28 (82.4%)	79 (41.6%)	<0.001
Necrotizing Enterocolitis [Y]	6 (17.6%)	10 (5.3%)	0.02
Intraventricular hemorrhage [Y]	7 (20.6%)	10 (5.3%)	0.006
Any surgical intervention [Y]	1 (2.9%)	15 (7.9%)	0.477
Evidence of fluid overload* [Y]	1 (2.9%)	5 (2.6%)	1.000
Multiple seizure* [Y]	8 (23.5%)	38 (20%)	0.639
Nephrotoxic drugs [Y]$	34 (100%)	188 (98.9%)	1.000
Furosemide [Y]$	6 (17.6%)	10 (5.3%)	0.02
Caffeine [Y]	8 (23.5%)	54 (28.4%)	0.557
Inotropes [Y]$	33 (97.1%)	129 (67.9%)	<0.001
Mean arterial pressure*#	48 (40 - 56)	46 (42 - 53.5)	0.959
IV fluid intake (ml/kg/d)*#	60 (60 - 72.5)	60 (60 - 70)	0.817
Serum creatinine ≥0.98 mg/dl [Y]$	33 (97.1%)	190 (100%)	0.152
Urine output <1.32 ml/kg/hr [Y]$	30 (88.2%)	113 (59.5%)	0.001
Serum urea (mg/dl)*#	46.5 (39 - 63.5)	40 (34.5 - 52)	0.006
Serum sodium (meq/L)*#	135 (132 - 140)	135 (132 - 139)	0.89
Serum potassium(meq/L)*#	5.5 (5.2 - 6)	5.3 (5 - 5.4)	0.003
Hb (g/L)*#	15.4 (13.2 - 16.3)	16.2 (15.2 - 17.7)	0.006
Serum pH*#	7.19 (7.00 - 7.26)	7.28 (7.21 - 7.32)	0.001
STARZ score*#	77 (71 - 84)	64 (50 - 77)	<0.001
No. of days to AKI#	1 (1 - 2)	1 (1 - 3)	0.017
NICU stay (days)#	7 (3 - 11)	12 (6 - 23)	0.001
Death [Y]	30 (88.2%)	9 (4.7%)	<0.001

#reported as median (IQR); for others as proportion
*First 12 hours post admission in NICU; ^Baseline maternal characteristics
$Variables used in the STARZ score
IQR: Interquartile range; AKI: Acute Kidney Injury; NICU: Neonatal Intensive Care Unit; hr: hour; mg: milligram; dl: decilitre; cm: centimetre; ml: millilitre; L: Liter; Hb: haemoglobin; g: gram; IV: Intravenous; hr: hour; kg: kilogram; d: day; meq: milliequivalent; PPV: Positive pressure ventilation; Y: Yes

- Nephrotoxic drugs included Aminoglycoside or Vancomycin or Colistin or Amphotericin B
- Inotropes included Dopamine or Dobutamine or Epinephrine or Norepinephrine
- Significant cardiac disease included PDA: Patent ductus arteriosus; PPHN: pulmonary hypertension of the newborn; VSD: Ventricular septal defect; shock
- Severe peripartum event included cord prolapsed, precipitate labour, abruption

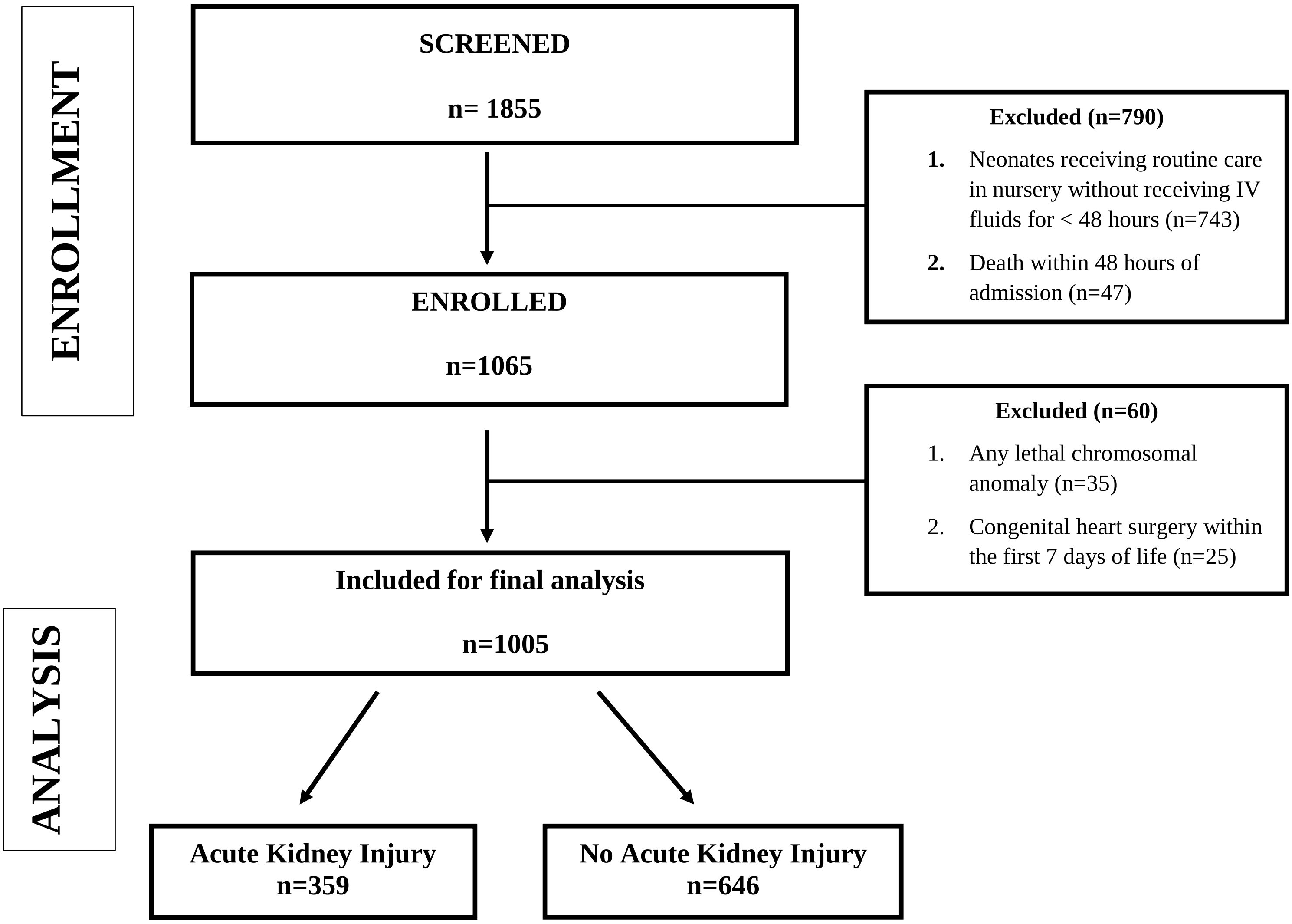
ENROLLMENT
SCREENED
n= 1855
Excluded (n=790)
1. Neonates receiving routine care in nursery without receiving IV fluids for < 48 hours (n=743)
2. Death within 48 hours of admission (n=47)
ENROLLED
n=1065
Excluded (n=60)
1. Any lethal chromosomal anomaly (n=35)
2. Congenital heart surgery within the first 7 days of life (n=25)
ANALYSIS
Included for final analysis
n=1005
Acute Kidney Injury
n=359
No Acute Kidney Injury
n=646

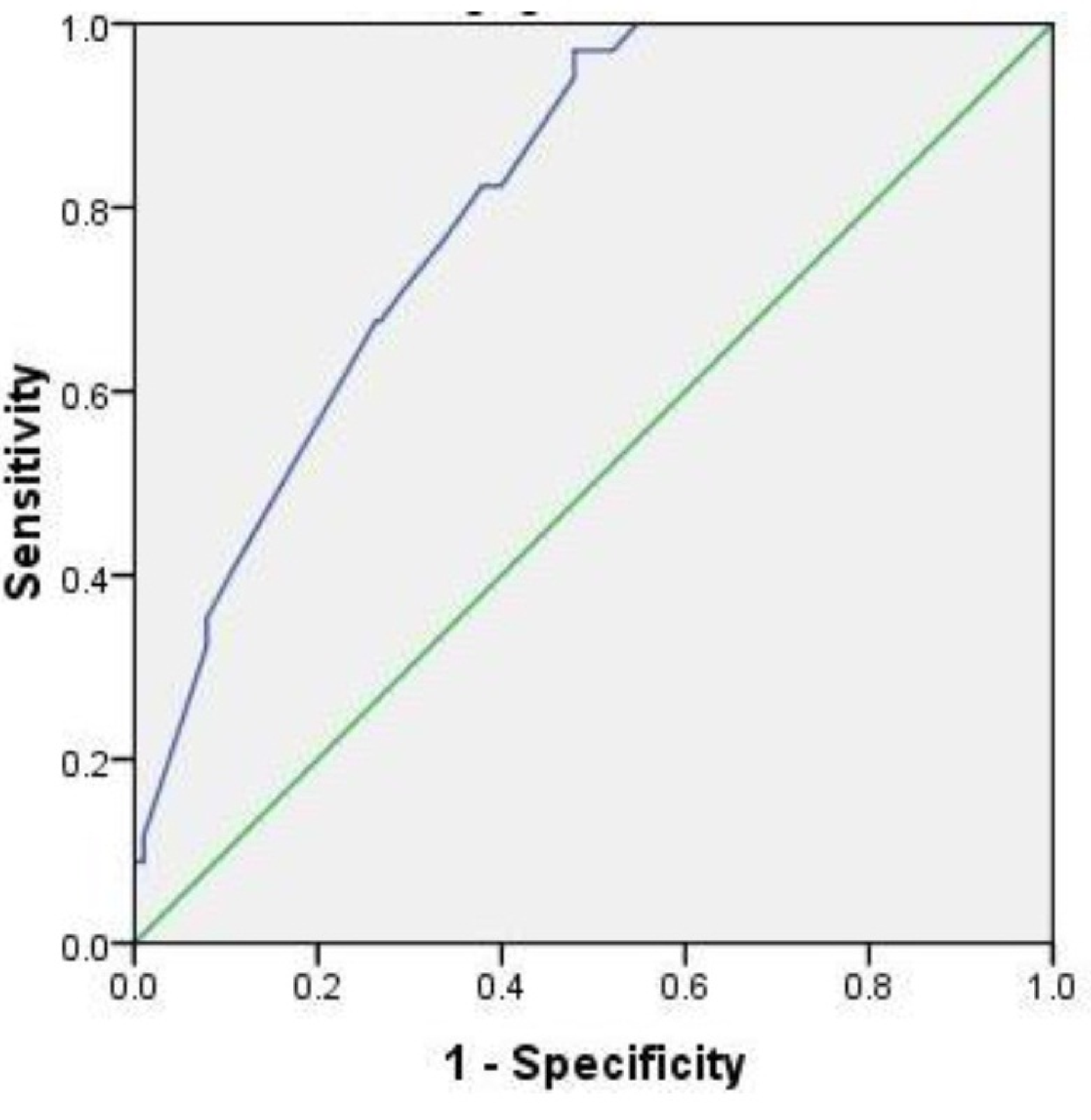

Area under the ROC curve: 0.804 (0.738 – 0.870); p<0.001
